# Mild hypothermia in rat with acute myocardial ischaemia‐reperfusion injury complicating severe sepsis

**DOI:** 10.1111/jcmm.16649

**Published:** 2021-05-31

**Authors:** Zhuyun Qin, Shixuan Shen, Kaiyong Qu, Yu Nie, Haitao Zhang

**Affiliations:** ^1^ State Key Laboratory of Cardiovascular Disease National Center for Cardiovascular Disease Chinese Academy of Medical Sciences and Peking Union Medical College Fuwai Hospital Beijing China; ^2^ Zhengzhou University People's Hospital Henan Provincial People's Hospital Zhengzhou China

**Keywords:** lipopolysaccharide, myocardial ischaemia‐reperfusion injury, severe sepsis, therapeutic mild hypothermia

## Abstract

Myocardial ischemia‐reperfusion injury (MIRI) with concurrent severe sepsis has led to substantial mortality. Mild hypothermia (MHT) has been proved to have a therapeutic effect in either MIRI or severe sepsis, which suggests it might be beneficial for MIRI complicating severe sepsis. In this study, Sprague‐Dawley rats with MIRI complicating severe sepsis were allotted in either MHT (33 ± 0.5°C) group or normothermia (NT, 37 ± 0.5°C) group; as control, rats receiving sham surgery and normal saline were kept at NT. After 2h of temperature maintenance, blood and heart tissue were acquired for detections. Lactate dehydrogenase (LDH) and MB isoenzyme of creatine kinase (CK‐MB) in blood, triphenyl tetrazolium chloride and Evans blue staining, hematoxylin and eosin staining for myocardium were employed to detect myocardial damage. Tumor necrosis factor (TNF)‐α and caspase‐3 was performed by immunohistochemistry to exam myocardial inflammation and apoptosis. Detection of NADPH oxidase (NOX) 2 was for myocardial oxidative stress. In MHT group, systolic blood pressure was improved significantly compared with NT group. Myocardial infarct size, morphological change, LDH and CK‐MB levels were attenuated compared to NT group. Moreover, less expressions of TNF‐α, caspase‐3 and NOX2 in MHT group were presented compared with NT group. MHT showed cardioprotection by improving cardiac dysfunction, reducing myocardial infarct size and attenuating myocardial injury, inflammation, apoptosis and oxidative stress.

## 1 INTRODUCTION

Both cardiovascular diseases and sepsis are major causes of global burden of disease. Patients with acute myocardial infarction (AMI) complicating sepsis are commonly witnessed in intensive care units. AMI complicated with severe sepsis can accelerate death and thus caused substantial mortality.^1^ The sudden blockade of coronary arteries decreases the blood supply of involved myocardial regions and generally causes AMI. Meanwhile, blood restoration can exacerbate the damage to heart, leading to myocardial ischaemia‐reperfusion injury (MIRI)^2^; without proper treatments, patients are susceptible to get infected and subsequently develop into sepsis. Severe sepsis is manifested by multiple organ dysfunction syndrome due to host's response to endotoxin.^3^ Unlike MIRI that causes tissue hypoxia, sepsis‐induced organ damage might be associated with impaired oxygen utilization, also referred to as ‘cytopathic hypoxia’.^4^ Thus, the heart may suffer from both insufficient oxygen supply and impaired oxygen utilization in patients with MIRI complicating severe sepsis, which is a conundrum for clinicians.

Mild hypothermia (MHT) has been recognized beneficial in either MIRI or sepsis. It decreases myocardial infarct size, prevents cardiac apoptosis, improves cardiac function and alleviates myocardial morphological change.^5^, ^6^ Albeit the popular recognition that MHT weakens body immune system, whether hypothermia is protective in infection is yet disputed, for evidence has shown MHT attenuates inflammation and oxidative stress in animals with severe sepsis.^3^, ^7^, ^8^ However, due to the intricate mechanisms of the co‐existence of MIRI and severe sepsis, none contributions have been found to study treatment on MIRI complicating severe sepsis.

Herein, based on the presupposition of the salubrious effects of MHT, we presumed MHT is effective in treating MIRI with severe sepsis and firstly applied MHT into the disease model of Sprague‐Dawley (SD) rat with MIRI combining severe sepsis and substantiated the beneficial effects of MHT through improving the systemic and cellular symptoms. MHT can be a potential treatment for MIRI complicating severe sepsis, which lies a foundation of more profound studies in the future.

## 2 MATERIALS AND METHODS

### 2.1 Animals and experimental protocol

The experimental protocol was approved by the local ethics committee, and the study was in consistent with guidelines in the Guide for the Care and Use of Laboratory Animals. Rats were purchased from Vital River Laboratory Animal Technology Co., Ltd.

**FIGURE 1:**
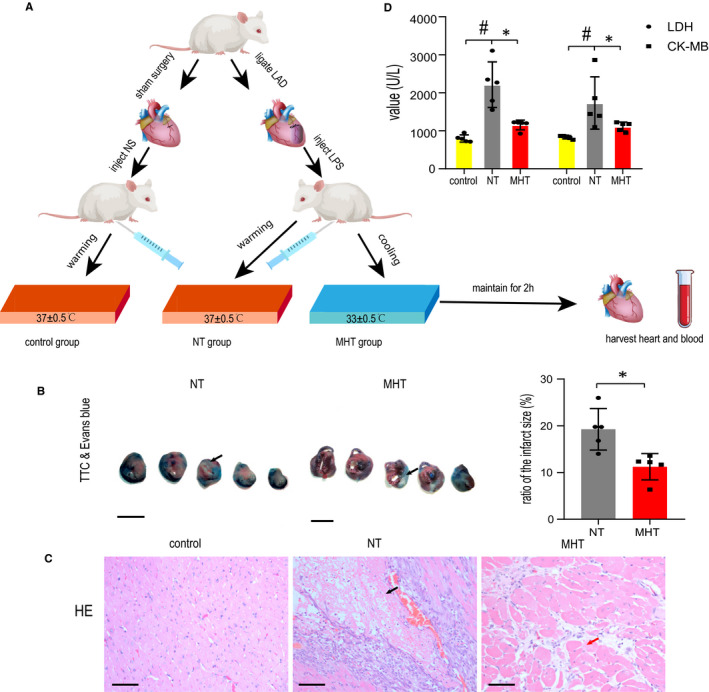
Schematic diagram of experimental protocol and myocardial damage in each group (n = 5). A, Schematic diagram of experimental protocol. B, Example pictures of TTC and Evans blue staining for myocardial infarct size in NT group and mild MHT group, the arrow shows the infarct area (scale bars:10 mm). Data are expressed as mean ±SD (^*^ *P* < .05 vs NT). C, Representative pictures of HE staining for myocardial morphological change in each group, black arrow shows the necrotic area and scarlet arrow shows myocardial oedema (x200, scale bars: 50 μm). D, Serum LDH and CK‐MB levels in each group. Data are expressed as mean ±SD (^*^ *P* < .05 vs NT, ^#^ *P* < .05 vs control). Abbreviations: CK‐MB, MB isoenzyme of creatine kinase; HE, haematoxylin and eosin; LAD, left anterior descending branch of coronary artery; LDH, lactate dehydrogenase; MHT, mild hypothermia; NS, normal saline; NT, normothermia; TTC, triphenyl tetrazolium chloride

A total of 15 SD rats (male, weight: 350‐450 g, n = 5) were randomly assigned into MIRI with severe sepsis in normothermia (NT, 37 ± 0.5°C) or MHT (model, 33 ± 0.5°C) group; for control, rats received sham surgery and normal saline (NS) were in NT (Figure 1A). MIRI was achieved, and the related electrocardiogram (ECG) and haemodynamic values were recorded according to the method in previous studies.^9^, ^10^ Lipopolysaccharide (LPS, 15 mg/kg, ip, *Escherichia coli*, O111:B4, Sigma‐Aldrich) was injected when reperfusion commenced while control group received a tantamount dosage of NS. During the surgery, body temperatures of the rats were maintained within a range of 37 ± 0.5°C.

Mild hypothermia was introduced immediately after the injection of LPS. Cooling was maintained for whole 2 hours at 33 ± 0.5°C, and the target temperature reached within 30 minutes. NT group received the same procedure. After 2 hours, heart and blood samples were acquired. The heart was used to detect infarct size, morphological change, inflammation, apoptosis and oxidization. The blood was used to test levels of lactate dehydrogenase (LDH) and MB isoenzyme of creatine kinase (CK‐MB) according to the preceding study.^11^

### 2.2 Triphenyl tetrazolium chloride (TTC) and Evans blue staining

Procedures have been described previously.^9^ The ratio was infarcted areas to the overall region ×100%.

### 2.3 Haematoxylin and eosin (HE) staining

Procedures have been described previously.^11^ Myocardial injury was based on the infiltration of inflammatory cells and myocardial morphological change.

### 2.4 Immunohistochemistry

Procedures have been described previously.^11^ The sections were incubated with anti‐TNF‐α (1:200, ab220210, Abcam) and anti‐caspase‐3 (pro‐caspase‐3, 1:200, ab184787, Abcam) antibodies. In each group, 5 heart tissues were used for immunohistochemistry detection. At least 3 random sights were analysed in each tissue. TNF‐α and caspase‐3 positive cells were quantified by Image J (Version 1.52q, National Institutes of Health) and determined by the ratio of positive cells to overall cells.

### 2.5 Western blot (WB) analysis

Procedures have been described previously.^2^ Proteins were extracted from myocardial tissue and homogenized with phosphate buffer solution and Tween. The primary antibodies were against NADPH oxidase 2 (NOX2, 1:800, 19013‐1‐AP, Proteintech). The band densities were normalized to β‐actin (1:2000, ab8226, Abcam).

### 2.6 Statistical analysis

Student's *t* test was used to compare difference between two groups, and one‐way ANOVA was for comparison among more than two groups. Differences were mean ± SD and considered statistically significant at *P* < .05.

## 3 RESULTS AND DISCUSSION

The classic animal models of MIRI have been used in numerous experiments throughout a long‐time span from 1980s to today.^9^, ^12^, ^13^, ^14^ Thanks to endotoxin that usually generates severe sepsis, lipopolysaccharide (LPS)‐induced sepsis in animals is widely recognized.^15^ In this study, to mimic clinical condition of MIRI complicating with severe sepsis, we combined MIRI and LPS‐induced sepsis in rats. Myocardial impairment of the rats caused by MIRI and severe sepsis was achieved conclusively through the change in haemodynamics, the patent myocardial infarct size, morphological change as well as the incremental expressions of TNF‐α, caspase‐3 and NOX2.

### 3.1 Mild hypothermia improved systolic and diastolic function

Haemodynamics was monitored for cardiac function. Table 1 summarized haemodynamic parameters of all experimental rats. Baselines of heart rate (HR), min dp/dt, diastolic pressure (DBP) and mean pressure (MAP) in each group were similar (n = 5), while baselines of max dp/dt in NT group and systolic pressure (SBP) in MHT group showed statistical significance compared to control (*P* < .05 vs. *P* < .05). This was probably due to the individual difference of the rats and the influences of anaesthesia. Data of the 2 hours of reperfusion were compared to the baselines and among groups. Left ventricular (LV) systolic function was assessed by max dp/dt, and diastolic function was assessed by min dp/dt. Values remained at a stable range at all stages in control group. Compared with baselines, in NT and MHT group, all values diminished during ischaemic status; at the 2 hours of reperfusion, max dp/dt, min dp/dt, SBP, DBP and MAP elevated in both NT group and MHT group. All values showed no significances compared among groups. However, tendency shows values but HR in MHT groups improved more significantly compared with NT group (*P* > .05). HR decreased in MHT group compared with NT group (*P* > .05) at the 2 hours of reperfusion, suggesting an improved myocardial contractility.

**TABLE 1:** Haemodynamic parameters: heart rate, cardiac systolic function, diastolic function and blood pressure (n = 5, mean ± SD)

		Control	NT	MHT
Heart rate (BPM)	Baseline	458.83 ± 13.89	420.59 ± 54.51	399.07 ± 59.28
	Ischaemia/sham	446.45 ± 3.37	391.02 ± 66.45	383.17 ± 33.25*
	Reperfusion 2 h	433.93 ± 27.52	457.74 ± 32.77	396.63 ± 39.93
Max dP/dt (mm sHg/s)	BASELINE	2872.98 ± 184.91	2437.92 ± 225.09*	2748.54 ± 581.84
	Ischaemia/sham	2592.79 ± 225.35	1806.00 ± 384.32*	2033.03 ± 771.24
	Reperfusion 2 h	2701.06 ± 128.72	2615.43 ± 741.91	4170.43 ± 2171.29
Min dP/dt (mm Hg/s)	Baseline	−2328.23 ± 234.39	−2075.96 ± 101.52	−2138.30 ± 670.48
	Ischaemia/sham	−1919.09 ± 248.52**	−1289.32 ± 185.73* ^,^ **	−1499.75 ± 552.15
	Reperfusion 2 h	−2656.64 ± 83.89	−−2151.56 ± 683.17	−3192.78 ± 1511.00
Systolic pressure (mm Hg)	Baseline	95.90 ± 2.84	103.07 ± 12.42	110.64 ± 8.34 *
	Ischaemia/sham	91.10 ± 0.79**	65.59 ± 9.06 * ^,^ **	83.90 ± 19.23**
	Reperfusion 2 h	92.34 ± 0.76**	102.89 ± 11.07	118.89 ± 10.01
Diastolic pressure (mm Hg)	Baseline	85.72 ± 5.23	92.49 ± 11.66	96.48 ± 16.75
	Ischaemia/sham	74.50 ± 2.87**	57.75 ± 12.25**	69.51 ± 27.13
	Reperfusion 2h	71.45 ± 0.45**	86.00 ± 11.24	90.57 ± 27.13
Mean pressure (mm Hg)	Baseline	90.85 ± 3.90	98.17 ± 13.00	103.24 ± 12.55
	Ischaemia/sham	83.84 ± 1.32**	60.91 ± 11.12**	78.61 ± 23.39
	Reperfusion 2 h	83.11 ± 0.61**	95.14 ± 12.16	99.36 ± 14.79

Abbreviations: MHT, mild hypothermia; NT, normothermia.

* *P* < .05 vs control.

** *P* < .05 vs baseline.

Whether MHT improves cardiac function remains a question. Although some previous investigations found no significant change in haemodynamics, or even an impaired cardiac diastolic function, it is affirmed MHT leads to stronger myocardial contractility.^16^, ^17^ Our study proved MHT improves myocardial contractility and presented better systolic and diastolic function in haemodynamics, though without statistical difference. This can correlate to scant samples or insufficient duration of MHT. In the study of Huang and colleagues,^10^ MHT improved both LV systolic and diastolic function (*P* < .001). In their study, MHT was being monitored for 4 hours, which was longer than 2 hours of ours. As for samples, 6 rats were used in each group in theirs while 5 were in ours. However, an animal study in vivo showed MHT did not exert positive inotropic effect.^18^ Thus, MHT on cardiac functional change of MIRI complicating severe sepsis needs more exploration.

### 3.2 Mild hypothermia reduced infarct size and myocardial damages

Mild hypothermia can reduce myocardial infarct size.^6^ Rather, Kanemoto et al^9^ proved the timing of initiate cooling is vital to myocardial salvage and cooling at reperfusion yielded the smallest infarct size. Identical to their study, cooling initiated at the time of reperfusion in ours, myocardial infarct areas in NT and MHT group were presented by TTC and Evans blue staining (Figure 1B). The result showed the ratio of myocardial infarct size to the overall region in MHT group decreased 10%‐20% compared to NT group (*P* < .05), which substantiated the effectiveness of MHT commenced at the time of reperfusion and offered an evidence on clinical treatment timing. Based on the infarct size, HE staining demonstrated less myocardial injury in the MHT group. In NT group, an obvious area of necrosis and a large number of inflammatory cells infiltrated in the interstitial areas can be seen. Meanwhile, in MHT group, inflammatory cells and erythrocytes assembled in the interstitial areas of left ventricle and myocardial oedema and slighter necrosis were observed (Figure 1C). These morphological changes of myocardium further suggested the cardioprotective effect of MHT.

When myocardial injury occurs, LDH and CK‐MB are released into peripheral circulation and the elevation is correlated with the degree of myocardial lesion. In our study, LDH and CK‐MB both elevated in all groups (Figure 1D). In NT group, LDH and CK‐MB elevated significantly compared to control (*P* < .05 vs. *P* < .05). Albeit LDH and CK‐MB in MHT group elevated as well, they were insignificant compared to control (*P* > .05 vs. *P* > .05). Compared with NT group, less elevations of LDH and CK‐MB displayed in significance in MHT group (*P* < .05). These altogether results revealed that MHT presented cardioprotection of myocardial injury.

### 3.3 Mild hypothermia palliated myocardial inflammation, apoptosis and oxidative stress

**FIGURE 2:**
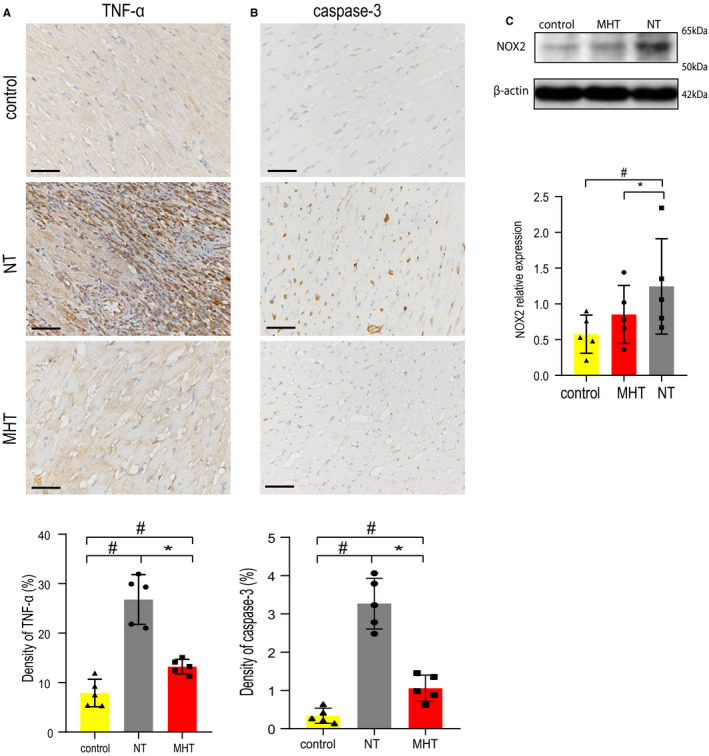
Cardiac inflammatory, apoptosis and oxidative changes in each group (n = 5). A, Inflammation in myocardium, representative pictures of TNF‐α expression (x200, scale bars: 50 μm). Data are expressed as mean ±SD (^*^ *P* < .05 vs NT, ^#^ *P* < .05 vs control). B, Apoptosis in myocardium, representative pictures of caspase‐3 expression (x200, scale bars: 50 μm). Data are expressed as mean ±SD (^*^ *P* < .05 vs NT, ^#^ *P* < .05 vs control). C, Representative picture of Western blot for NOX2 expression. Data are shown as mean ±SD (^*^ *P* < .05 vs NT, ^#^ *P* < .05 vs control). Abbreviations: MHT, mild hypothermia; NOX2: NADPH oxidase 2; NT, normothermia; TNF‐α, tumour necrosis factor‐α

Proinflammatory cytokines, which relate to inflammation and cell apoptosis, can be released to the damaged myocardial area rapidly after the cardio being attacked. Inflammation was assessed by TNF‐α (Figure 2A). Higher TNF‐α expressed in NT and MHT group than control (*P* < .05 vs *P* < .05). Moreover, TNF‐α expressed decidedly less in MHT group than in NT group (*P* < .05). MHT can inhibit inflammatory response in either MIRI or sepsis in other experimental studies, which declines inflammatory cytokines and preserve organ functions.^18^, ^19^ In our study, TNF‐α was shown less in the MHT group, suggesting the anti‐inflammatory response effect of MHT in MIRI with sepsis.

Pro‐caspase‐3 is an inactive form of caspase‐3 and is a sign of caspase‐3 activation. Studies show pro‐caspase‐3 can protect cell from apoptosis.^20^, ^21^ In this study, apoptosis was assessed by pro‐caspase‐3 (Figure 2B). Though not strong, caspase‐3 was presented higher expression in NT group than control (*P* < .05). The expression in MHT was also observed higher compared to control (*P* < .05). Accordingly, caspase‐3 was expressed less in MHT group than in NT group (*P* < .05). Thus, the results illustrated MHT also has an effect of anti‐myocardial apoptosis.

Superoxide dismutase (SOD) and malondialdehyde (MDA) are major oxidative products. Cheng et al^2^ found hypothermia decreases SOD and MDA and shows a cardioprotective effect against MIRI. Additionally, MHT reduces organ SOD and MDA in sepsis.^8^ NOX2 is proved to be a target in cardiac oxidative stress reaction, which generates reactive oxygen species (ROS), such as SOD and MDA, and elevated ROS leads to oxidative stress.^22^ Therefore, elevation of NOX2 can reveal existence of oxidative stress reaction. NOX2 expression was performed in WB analysis (Figure 2C). In our study, MHT demonstrated a mitigation of oxidative stress reaction. An obvious more NOX2 expression in NT group than control was presented (*P* < .05), and the expression in MHT group was similar with control (*P* > .05). Simultaneously, expression of NOX2 was lower in MHT group compared to NT group (*P* < .05). These findings suggest MHT can attenuate myocardial oxidative stress in rat with MIRI combining severe sepsis.

In addition, though our study proved the cardiac beneficial effect of MHT in MIRI with severe sepsis, the mechanism remains unclear yet. Nonetheless, imbalanced energy consumption and oxygen supply have been observed in both MIRI and sepsis. According to studies, preserving the integrity of mitochondria and its function can alleviate symptoms of either MIRI or sepsis.^16^, ^18^ Thus, a possible explanation of MHT ameliorating cardiac damage in either MIRI or severe sepsis is through lowering body metabolism and affecting on mitochondria. Hence, further studies can be carried out on mitochondria and the related molecular pathways. Even though whether MHT is effective in human with MIRI complicating severe sepsis is under discovery, the results in this study provided a base that MHT is promising in improving the cardiac outcomes.

## 4 CONCLUSION

Both MIRI and sepsis can injure heart by generating myocardial inflammation, apoptosis and oxidative stress reaction in the host, leading to cardiac structural and functional damage. In our study, MHT showed a cardioprotective effect from aspects of haemodynamic change, myocardial infarct size, myocardial injury, inflammation, apoptosis and oxidative stress in rats with MIRI combining severe sepsis.

## 5 LIMITATIONS

The LPS‐induced sepsis differs from sepsis caused by microorganisms clinically. Other treatments and rewarming process were not incorporated in this study. Moreover, interspecies difference may limit the transfer from our results to mankind and sensibility of each rat to MHT was distinct.

## CONFLICT OF INTERESTS

The authors confirm that there are no conflicts of interest.

## AUTHOR CONTRIBUTIONS

**Zhuyun Qin:** Conceptualization (lead); Data curation (lead); Formal analysis (lead); Investigation (lead); Methodology (lead); Validation (lead); Writing‐original draft (lead). **Shixuan Shen:** Methodology (supporting); Validation (supporting). **Kaiyong Qu:** Methodology (supporting). **Yu Nie:** Writing‐review & editing (equal). **Haitao Zhang:** Conceptualization (lead); Project administration (lead); Supervision (lead); Validation (equal); Writing‐review & editing (lead).

## Data Availability

The data that support the findings of this study are available from the corresponding author upon reasonable request.
